# Larger deep white matter hyperintensity volume correlates with more severe social behavioral changes in patients with subacute ischemic stroke

**DOI:** 10.3389/fnagi.2023.1259690

**Published:** 2023-11-22

**Authors:** Jian-Feng Qu, Hui-Hong Hu, Jian-Fei Liu, Yue-Qiong Zhou, Wei-Yang Cheng, Lin Shi, Yi-Shan Luo, Lei Zhao, Yang-Kun Chen

**Affiliations:** ^1^Department of Neurology, The Tenth Affiliated Hospital of Southern Medical University (Dongguan People’s Hospital), Dongguan, Guangdong, China; ^2^Intelligent Brain Imaging and Brain Function Laboratory (Dongguan Key Laboratory), Dongguan People’s Hospital, Dongguan, Guangdong, China; ^3^Community Health Service Center of Dongguan Dongcheng, Dongguan, Guangdong, China; ^4^Faculty of Neurology, Graduate School of Southern Medical University, Guangzhou, Guangdong, China; ^5^Faculty of Neurology, Graduate School of Guangdong Medical University, Zhanjiang, Guangdong, China; ^6^Department of Imaging and Interventional Radiology, The Chinese University of Hong Kong, Shatin, Hong Kong SAR, China; ^7^BrainNow Research Institute, Shenzhen, Guangdong, China

**Keywords:** ischemic stroke, white matter hyperintensities, brain structure volumetry, mRS score, social behavior

## Abstract

**Background:**

Changes in social behavior can occur after ischemic stroke. We aimed to investigate the potential correlations between neuroimaging variables and changes in social behavior in patients who experienced subacute ischemic stroke.

**Methods:**

We prospectively screened patients with first-ever ischemic stroke. Three months after the index stroke, changes in patients’ social behavior were investigated by the Frontal Behavioral Inventory (FBI), which consists of both deficit and positive groups of behaviors. The protocol of brain magnetic resonance imaging (MRI) including the baseline MRI at the acute stage and additional MRI with three-dimensional T1-weighted imaging on follow-up. Using these MRI scans, we assessed the acute infarction and the volumes of various brain structures by an automatic volumetry tool.

**Results:**

Eighty patients were enrolled. In univariate analyses, patients with deficit behavioral changes had more left cortical infarction (*r* = 0.271, *p* = 0.015), Cholinergic Pathways Hyperintensities Scale scores (*r* = 0.227, *p* = 0.042), DWMH volumes (*r* = 0.349, *p* = 0.001), and modified Rankin Scale (mRS) scores (*r* = 0.392, *p* < 0.001). Patients with positive behavioral changes had more frequency of men (*r* = 0.229, *p* = 0.041) and a history of hypertension (*r* = 0.245, *p* = 0.028). In multiple stepwise linear regression models, after adjusting for age, deep WMH volumes (*β* = 0.849, 95% confidence interval = 0.352–1.346, *p* = 0.001) and mRS scores on follow-up (*β* = 1.821, 95% confidence interval = 0.881–2.76, *p* < 0.001) were significantly correlated with deficit behavioral changes (*R*^2^ = 0.245).

**Conclusion:**

Larger deep WMH volumes and poorer mRS scores on follow-up were significantly correlated with deficit behavioral changes in patients with subacute ischemic stroke.

## Introduction

Social cognition (SC) is a fundamental neurocognitive capability that refers to the perception, processing, and interpretation of social information (Henry et al., 2016). SC is also critical for the understanding of oneself and others, as well as of societal norms and procedures. Disturbances of SC can exist in many neuropsychiatric or chronic neurological disorders; more over, they also can happen in patients with stroke (Henry et al., 2016). Impairments in SC have been correlated with poorer outcomes, including quality of life, mental health problems, unemployment, and loneliness (Phillips et al., 2010). Even in stroke patients with good physical recovery, SC impairments may hinder reintegration to everyday life (Qu et al., 2022a,b).

Adams et al. (2019) reported that SC deficit in stroke can be identified in four core domains: theory of mind, social perception, affective empathy, and social behavior. Social behavior refers to how a person performs during interactions with other people (Santamaría-García et al., 2022). Besides with social behavior, long-term impairments in SC are even present in mildly affected stroke patients; and these impairments may perplex such patients permanently (Nijsse et al., 2019).

The exact pathophysiological mechanism of SC impairment has not been identified. Gunaydin et al. (2014) demonstrated that the optogenetic control of cells that specifically contribute to projections from the ventral tegmental area to the nucleus accumbens modulate the social behavior. In an animal experiment, the researchers found that in mice with striatal cholinergic interneurons ablation, the normal pattern of social exploration was replayed continuously and, arising compulsive social behavior (Yanina et al., 2017). These specific neurons often form specific structure or circuit. Chaudhury et al. (2013) reported that, in the ventral tegmental area, dopamine neurons in the brain’s reward circuit have a crucial role in mediating stress responses, including determining susceptibility versus resilience to social-stress-induced behavioral abnormalities. Kana et al. (2021) found in patients with Alzheimer’s dementia that the medial septum/vertical diagonal band of Broca and nucleus basalis magnocellularis cholinergic neurons play distinct roles in social interaction and social recognition memory (Kana et al., 2021). As the intact SC related to different scope of neural structure, from neurotransmitter to structural brain abnormalities, involve in the extensive white matter may contribute to changes in SC (Wang and Olson, 2018).

Several factors might contribute to the disturbance of social behavior after stroke. A previous study revealed that the severity of SC impairments is determined mainly by demographic factors (Adams et al., 2020). However, the putative risk factors of social behavioral changes in patients with stroke are not well understood, especially in relation to neuroimaging features. Hence, the present study tried to explore the clinical and neuroimaging factors which may correlate with changes in social behavior in patients with ischemic stroke.

## Methods

### Participants and setting

Patients with first-ever acute ischemic stroke in Division I, Department of Neurology, The Tenth Affiliated Hospital of Southern Medical University (Dongguan People’s Hospital), were recruited between 1 July 2021 and 30 December 2022. Acute ischemic stroke was defined according to an acknowledged criterion (Powers et al., 2019).

The inclusion criteria were: (1) older than 18 years; (2) a complete brain magnetic resonance imaging (MRI) examination was performed during admission; and (3) Because the moderate to severe neurological deficits may disturb the accuracy of the SC assessment, in order to assess the changes in social behavior more reliably, patients with mild stroke were screened, who met both of the following criteria: a National Institute of Health Stroke Scale (NIHSS) score ≤ 15 on discharge and a modified Rankin Scale (mRS) score ≤ 2 on discharge. Patients were excluded if they had: (1) a transient ischemic attack or no infarction on diffusion-weighted imaging; (2) an incomplete MRI or no MRI; (3) stroke complicated with hemorrhagic transformation; (4) severe stroke (NIHSS >15 or mRS > 2 on discharge); (5) stroke complicated with corona virus disease 2019 (COVID-19); (6) stroke complicated with severe comorbidities; (7) according to the pre-stroke and index medical records, a history of severe neuropsychological diseases or dementia; or (8) if the patient or their relatives refused to sign the informed consent form.

The study protocol was approved by the Ethics Committee of the Tenth Affiliated Hospital of Southern Medical University (Dongguan People’s Hospital). All patients provided informed written consent, as required by the Declaration of Helsinki.

#### Demographic and clinical data collection

Within the index admission, each patient’s clinical data were recorded. We also assessed the stroke severity by NIHSS and the stroke etiology by the Trial of ORG 10172 in Acute Stroke Treatment (TOAST) subtype system (Adams et al., 1993).

#### Imaging data

MRI acquisition was performed on a 3.0-T scanner (Skyra; Siemens, Erlangen, Germany). The protocol of brain MRI scans including the baseline MRI at the acute stage and additional MRI with three-dimensional T1-weighted imaging at 3 month ±2 weeks after the index stroke, for reducing the interference of acute ischemic lesions in the assessment of white matter hyperintensities (WMH) (Qu et al., 2018). The detailed MRI protocols have been described elsewhere (Qu et al., 2022a,b). The MRI at the acute stage was mainly used to assessed the acute infarction, while the additional MRI at 3 month was used to analyze the various brain volumetry, which including the WMH. The neuroimaging variables were as follows:

(1) Acute infarction. Both the site and volume of the acute infarct were recorded. We identified unilateral cortical, subcortical, and infratentorial lesions using diffusion-weighted imaging sequences at the acute stage. The infarct lesion of all patients was automatically segmented in the acute stage DWI images based on deep learning model. All follow-up MRI scans were analyzed using a fully automatic neuroanatomical volumetry tool (AccuBrain^®^, BrainNow Medical Technology Limited, Shenzhen, China).
(2) Brain volumetry. AccuBrain^®^ was used to quantify the volumes of various cortical, subcortical structures, ventricles, and WMH around 10 min. The WMH volumes included those of periventricular hyperintensities (PVH), deep WMH (DWMH), and whole WMH.
(3) Cortical cholinergic pathways. Lesions involving the cortical cholinergic pathways were evaluated by the hyperintensity signal on the MRI-T2 sequence, using the Cholinergic Pathways Hyperintensities Scale (Selden et al., 1998).
(4) Other MRI parameters. We also measured other brain abnormalities, including silent brain infarcts and enlarged perivascular spaces.

#### Follow-up examinations

We assessed changes in the social behavior status of patients using the Frontal Behavioral Inventory (FBI) on 3 months after the index stroke (Kertesz et al., 1997), by a trained doctor who was blinded to the MRI data. The FBI is a 24-item questionnaire that can be used to describe and quantify behavioral and personality changes in patients with not only different forms of dementia, but also stroke. The FBI items consist of deficit and positive groups of behaviors. The deficit behaviors comprise apathy, aspontaneity, indifference, inflexibility, concreteness, personal neglect, disorganization, inattention, loss of insight, logopenia, verbal apraxia, and alien hand. The positive behaviors comprise perseveration, irritability, excessive or childish jocularity, irresponsibility, inappropriateness, impulsivity, restlessness, aggression, hyperorality, hypersexuality, utilization behavior, and incontinence (Kertesz et al., 2000). The scoring of each item is compared with the premorbid state and is scored 0 when no changes exist, 1 when changes are mild, 2 when changes are moderate, and 3 when changes are severe, as gauged by a trained doctor. The maximum total score for either the deficit or positive group of behaviors is 36; the score is obtained by adding the scores of each of the 12 items (Benejam et al., 2009). A higher score reflects more severe behavioral changes (Malloy and Grace, 2005).

The other aspects of SC were also assessed:

Empathy. The status of empathy assessed by using the Chinese version of the Empathy Quotient (Zhao et al., 2018). The quotient comprises 60 questions, was broken down into tapping empathy items (40 questions) and 20 filler items. The total EQ score ranges from 0 to 80, with higher scores reflecting greater empathy (Zhao et al., 2018).
Social perception. Social perception was assessed by using the Chinese emotional facial expressions test (Wang et al., 2006). The test including a set of 30 photos of continuously changing emotional faces including the 6 basic emotions (happiness, anger, sadness, fear, disgust, and surprise) were formed by fusing them at the immovable proportions. Scoring consisted of the correct recognition and remote error recognition with each highest score was 120, respectively.

We also recorded the NIHSS score and mRS score on the interview.

### Statistical analysis

The characteristics of the participants are presented as means, or medians (interquartile range) and percentage. Correlations between FBI scores and variables were based on the Pearson’s or Spearman’s correlation analyses. After that, the deficit or positive score based on FBI assessment was, respectively, set as the dependent variable, the non-collinear variables (*r* < 0.6) with a smaller *p* value were set as independent variable in a linear regression analysis. A *p* value <0.05 was considered statistically significant.

## Results

**Figure 1:**
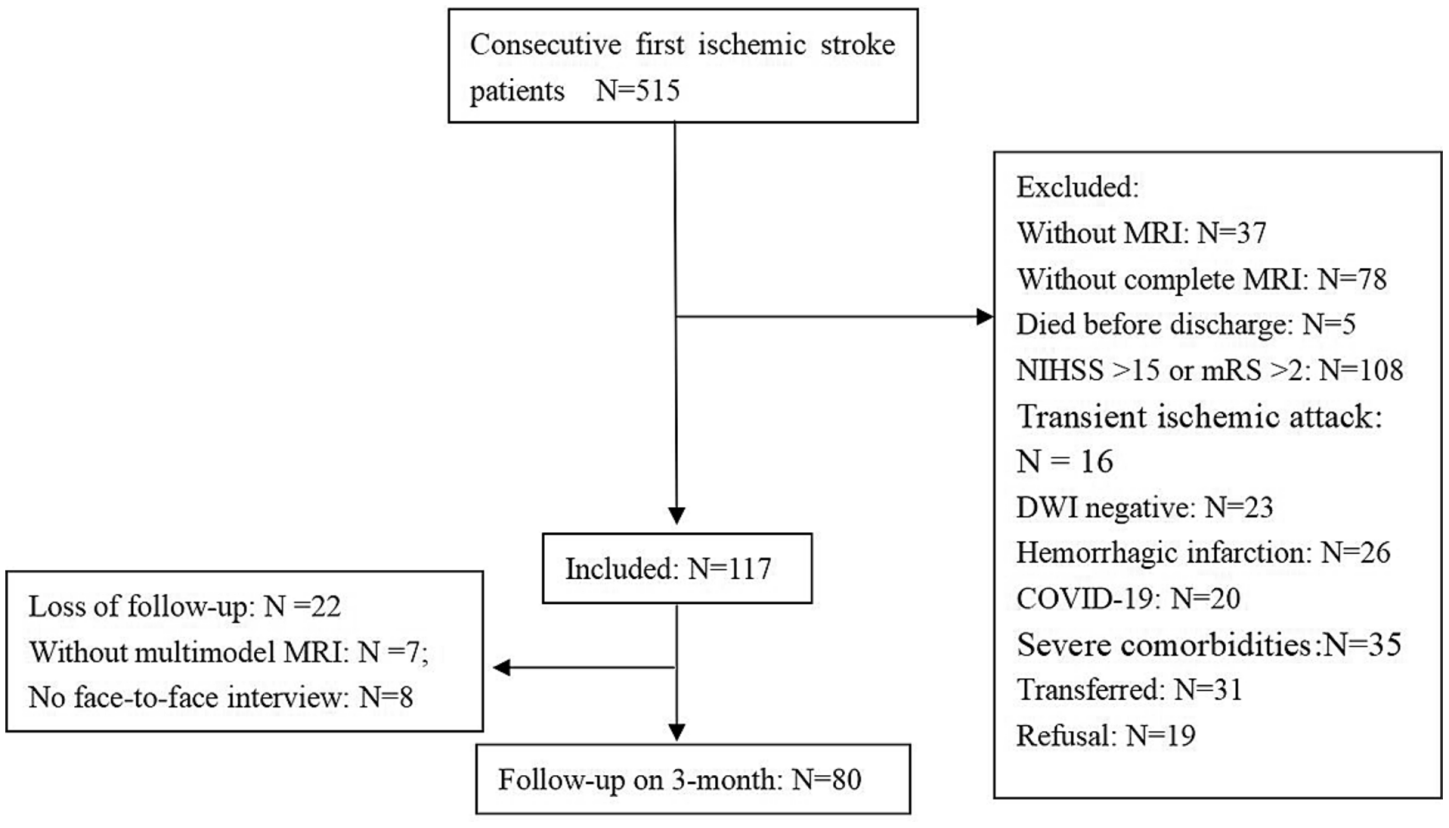
Flow chart showed the patient selection criteria.

During the study period, we had totally screened 515 patients with first-ever acute ischemic stroke. Finally, 80 patients were included in the analysis according to selection criteria (Figure 1).

The study population consisted of 51 (63.8%) men and 29 (36.2%) women (Table 1). The mean age was 60.53 ± 9.51 years and the NIHSS score on discharge was 1 (0–2).

**Table 1:** Demographics and clinical characteristics of the study sample.

Variables	*n* = 80	Deficit behavior		Positive behavior	
		*r*	*p*	*r*	*p*
Age (years) ^a^	60.53 ± 9.51	0.055	0.628	−0.026	0.819
Men ^b^	51 (63.8%)	−0.009	0.934	**0.229**	**0.041**
Hypertension ^b^	62 (77.5%)	0.091	0.424	**0.245**	**0.028**
Diabetes mellitus ^b^	19 (23.8%)	−0.066	0.561	−0.125	0.268
Atrial fibrillation ^b^	5 (6.3%)	0.049	0.669	−0.107	0.346
I.V. thrombolysis ^b^	11 (13.8%)	0.019	0.865	−0.095	0.402
Embolectomy ^b^	7 (8.8%)	0.129	0.253	−0.049	0.668
NIHSS on admission ^c^	2 (0–3)	0.196	0.081	−0.041	0.721
NIHSS on discharge ^c^	1 (0–2)	0.186	0.098	−0.017	0.884
TOAST ^b^		−0.214	0.057	−0.064	0.575
LAA	29 (36.3%)				
SAO	35 (43.8%)				
CE	9 (11.3%)				
SOC	1 (1.3%)				
SOU	6 (7.5%)				
Location of infarcts ^b^					
L-cortical	25 (31.3%)	**0.271**	**0.015**	0	1
R-cortical	18 (22.5%)	0.063	0.577	0.136	0.299
L- subcortical	33 (41.3%)	0.125	0.268	0.098	0.388
R- subcortical	24 (30%)	−0.017	0.879	−0.041	0.716
Infratentorial	20 (25%)	−0.104	0.359	−0.099	0.381
Infarct volume ^c^	0.985 (0.475–2.665)				
SBI ^c^	9 (2–17)	0.195	0.083	0.111	0.327
EPVS ^c^	1 (1–2)	−0.082	0.472	0.013	0.907
CHIPS ^a^	12.81 ± 14.77	**0.227**	**0.042**	−0.057	0.613
WMH volume ^c^	4.36 (1.85–10.48)	0.079	0.485	−0.032	0.778
PVH volume ^c^	3.695 (1.125–7.897)	0.047	0.677	−0.029	0.798
DWMH volume ^c^	0.82 (0.369–1.46)	**0.349**	**0.001**	−0.033	0.774
NIHSS on follow-up ^c^	0 (0–1)				
mRS on follow-up ^c^	1 (0–1)	**0.392**	**<0.001**	−0.014	0.903
Social behavior ^c^					
Deficit behavior	1 (1–3)	–	–	–	–
Positive behavior	0 (0–1)	–	–	–	–
Empathy ^c^	37.5 (30.5–48)	−0.199	0.076	0.064	0.573
Social perception ^c^					
Right recognition	37 (29.5–45)	−0.129	0.253	−0.119	0.294
Wrong recognition	50 (40–65)	−0.007	0.954	−0.005	0.966

^a^ Mean (SD), ^b^ n (%), ^c^ Median (IQR); LAA, large artery atherosclerosis; SAO, small artery occlusion; CE, cardioembolism; SOC, stroke of other causes; SUC, stroke of unknow causes; L, left; R, right; SBI, silent brain infarction; EPVS, Enlarged perivascular spaces; CHIPS, the Cholinergic Pathways HyperIntensities Scale; WMH, White matter hyperintensities; PVH, Periventricular hyperintensities; DWMH, Deep white matter hyperintensities; NIHSS, National Institute of Health Stroke Scale; mRS, modified Rankin Scale. The bold: a larger correlation coefficient with *p* value < 0.05.

The results of other SC on follow-up were as follows (mean [range]): empathy, 37.5 (30.5–48); facial expression right recognition, 37 (29.5–45); facial expression remote wrong recognition, 50 (40–65). There were no significant correlations between neither deficit or positive behavioral changes and the above other aspects of social cognition.

### The relationships between clinical and neuroimaging variables and FBI

We analyzed the correlations between clinical and neuroimaging variables and both deficit and positive behavioral changes. The were no significant correlation between neither total (Supplementary Table) or unilateral cortical or subcortical structures volumes and FBI scores (Table 2 and Supplementary Table). As shown in Table 1, deficit behavioral changes were correlated with left cortical infarction (*r* = 0.271, *p* = 0.015), Cholinergic Pathways Hyperintensities Scale scores (*r* = 0.227, *p* = 0.042), DWMH volumes (*r* = 0.349, *p* = 0.001), and mRS (*r* = 0.392, *p* < 0.001) on follow-up. In contrast, deficit behavioral changes were not correlated with brain structure volumes or any pre-existing brain abnormalities other than DWMH volume.

**Table 2:** Correlation analysis between Frontal Behavioral Inventory scores and unilateral brain volumes.

Variables	Volume	Deficit behavior		Positive behavior	
		*r*	*p*	*r*	*p*
Hippocampus -L	3.34 ± 0.33	0.039	0.734	0.064	0.570
Hippocampus R	3.47 ± 0.35	−0.028	0.805	0.123	0.275
Amygdala -L	1.85 ± 0.2	0.010	0.932	−0.040	0.726
Amygdala -R	1.86 ± 0.2	0.038	0.735	0.019	0.870
Lateral ventricle -L	17.07 ± 12.57	0.021	0.852	−0.159	0.159
Lateral ventricle -R	14.09 ± 10.12	0.134	0.237	−0.171	0.128
Inf -Lat- Vent -L	1.71 ± 0.68	0.143	0.206	−0.130	0.251
Inf -Lat-Vent -R	1.65 ± 0.74	0.109	0.338	−0.129	0.255
Thalamus -L	5.94 ± 0.58	−0.133	0.240	0.132	0.244
Thalamus -R	5.96 ± 0.74	−0.127	0.262	0.088	0.440
Caudate -L	3.47 ± 0.41	0.110	0.333	0.181	0.109
Caudate -R	3.37 ± 0.66	0.038	0.738	0.018	0.876
Putamen -L	5.11 ± 0.75	−0.108	0.341	0.080	0.479
Putamen -R	5.39 ± 0.8	−0.105	0.353	0.083	0.464
Pallidum -L	1.6 ± 0.24	−0.002	0.985	0.119	0.291
Pallidum -R	1.57 ± 0.74	0.033	0.768	0.118	0.299
Accumbens -L	0.54 ± 0.07	−0.058	0.607	0.214	0.057
Accumbens -R	0.53 ± 0.07	0.012	0.917	0.137	0.225
Hypothalamus -L	0.34 ± 0.04	−0.041	0.719	−0.078	0.490
Hypothalamus -R	0.36 ± 0.04	0.015	0.892	−0.084	0.461
Frontal Lobe -L	75.51 ± 8.38	0.027	0.812	0.157	0.163
Frontal Lobe -R	75.71 ± 8.21	0.045	0.694	0.135	0.231
Occipital Lobe -L	38.02 ± 4.82	−0.117	0.301	0.015	0.897
Occipital Lobe -R	33.91 ± 4.22	−0.083	0.462	−0.051	0.654
Temporal Lobe -L	51.68 ± 6.27	−0.016	0.889	0.021	0.856
Temporal Lobe -R	51.15 ± 6.06	−0.015	0.895	0.070	0.537
Parietal Lobe -L	37.8 ± 4.49	−0.080	0.481	0.012	0.914
Parietal Lobe -R	41.13 ± 5.19	−0.023	0.837	0.040	0.726
Cingulate Lobe -L	11.13 ± 1.82	0.070	0.540	−0.014	0.901
Cingulate Lobe -R	12.6 ± 1.92	0.043	0.705	0.084	0.459
Insular -L	6.32 ± 1.1	−0.061	0.590	0.100	0.380
Insular -R	6.79 ± 1	0.047	0.681	0.011	0.920

L, left; R, right; Inf -Lat- Ven, inferior angle of the lateral ventricle.

Positive behavioral changes were correlated with male sex (*r* = 0.229, *p* = 0.041) and a history of hypertension (*r* = 0.245, *p* = 0.028). In contrast, positive behavioral changes were not correlated with brain structure volumes or any pre-existing brain abnormalities (including DWMH volume).

### Linear regression analysis of the FBI

We performed distinct multiple stepwise linear regression models for the scores of deficit and positive behaviors. After adjusting for age, sex was correlated with positive behavioral changes (*β* = 0.692, 95% confidence interval = 0.029–1.355, *p* = 0.041). However, the *R*^2^ was only 0.021, indicating a relatively unstable relationship. The linear regression models for deficit behavioral changes are shown in Table 3. After adjusting for age, both DWMH volume (*β* = 0.849, 95% confidence interval = 0.352–1.346, *p* = 0.001) and mRS on follow-up (*β* = 1.821, 95% confidence interval = 0.881–2.76, *p* < 0.001) were significantly correlated with deficit behavioral changes (*R*^2^ = 0.245).

**Table 3:** Linear regression model analysis of deficit behavior scores.

Variables	Deficit behavior (*R*^2^ = 0.245)	
	*β*	*p*
L-cortical infarct	0.652 (−0.708–2.011)	0.343
CHIPS	0.003 (−0.042–0.048)	0.129
DWMH volume	0.849 (0.352–1.346)	0.001
mRS on follow-up	1.821 (0.881–2.76)	<0.001

adjusted for age. L, left; CHIPS, the Cholinergic Pathways Hyperintensities Scale; DWMH, Deep white matter hyperintensities; mRS, modified Rankin Scale.

## Discussion

**Figure 2:**
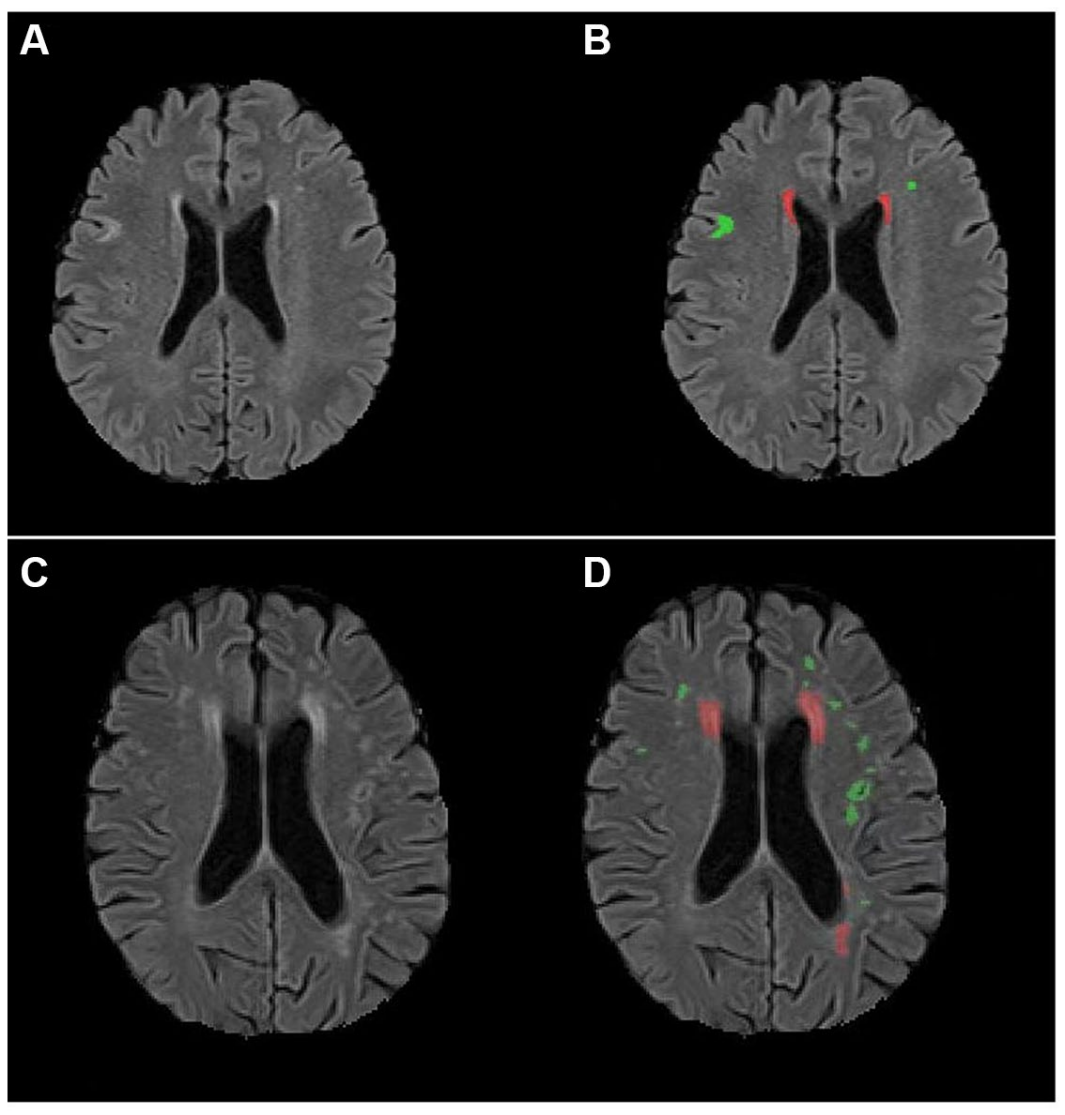
AccuBrain^@^ was used to quantify the total white matter hyperintensity volumes in patients with different changes in deficit social behaviors. **(A,B)** A 70-year-old man with subacute stroke had relatively mild changes in deficit social behaviors (Frontal Behavioral Inventory deficit score: 1). Panel **(A)** shows the original image of white matter hyperintensities. Panel **(B)** shows that the total deep white matter hyperintensity volume was 1.82 mL (marked with green) and the total periventricular hyperintensity volume was 1.71 mL (marked with red). **(C,D)** A 73-year-old woman with subacute stroke had relatively severe changes in deficit social behaviors (Frontal Behavioral Inventory deficit score: 13). Panel **(C)** shows the original image of white matter hyperintensities. Panel **(D)** shows that the total deep white matter hyperintensity volume was 3.42 mL (marked with green) and the total periventricular hyperintensity volume was 9.58 mL (marked with red).

We conducted a protocol of multimodal MRI and assessed changes in the social behavior of patients on 3 months post-stroke. The volumes of various subcortical, ventricular structures and WMH using the three-dimensional T1-weighted images were calculated by an automatic tool. Our main findings were that larger DWMH volumes and poorer mRS scores on follow-up were significantly correlated with more severe deficit behavioral changes in patients with subacute ischemic stroke (Figure 2).

White matter connectivity is essential for organized human behavior. Working together with gray matter to enable the large repertoire of human cognitive capacities (Filley and Fields, 2016). WMH can be found in stroke; and relate to many complications, such as decreased cortical thickness (Dickie et al., 2020), cognitive decline/ dementia (Tziaka et al., 2023), stroke-related mood disorders/depression (Kim et al., 2011; Zhang et al., 2023), physical recovery (Dai et al., 2022), and even functional status (Qu et al., 2022a,b). According to histopathologic research by Fazekas et al. (1993) WMH are usually distributed between PVH and confluent DWMH, and can be measured using a semiquantitative method known as the Fazekas grade scale. Benefit from the advanced neuroimaging analysis techniques, we now can precisely quantify the volumes of either PVH or DWMH, leading to more comprehensive insight to the relationship between WMH and stroke. Similar to a previous study (Kim et al., 2022), the median DWMH volume was 0.82 (0.369–1.46) ml in the presented study. Furthermore, our main finding was that larger DWMH volumes were significantly correlated with greater FBI deficit behavior scores. This finding suggests that more severe changes in deficit social behaviors are associated with larger DWMH volumes in subacute ischemic stroke. A relationship between WMH and SC impairment has also been observed in other studies. For example, a previous study investigating the contribution of WMH and cortical thickness to empathy found that cortical atrophy and WMH volume may be associated with empathy deficits in neurodegenerative and cerebrovascular diseases (Ozzoude et al., 2022). Moreover, Yang et al. (2022) reported that a higher WMH load is correlated with mild behavioral impairment in patients with normal cognitive function. The latent causality between WMH and SC impairment may be explained by pathological changes that are highlighted by modal magnetic resonance techniques. The extension of hyperintensities alongside the lateral ventricles, as a small rim or even a halo of PVH, appears to be associated with both a thin rim of subependymal gliosis and a wider, smooth band of white matter tissue, with reduced staining for myelin around the lateral ventricles (Fazekas et al., 1998).

**Figure 3:**
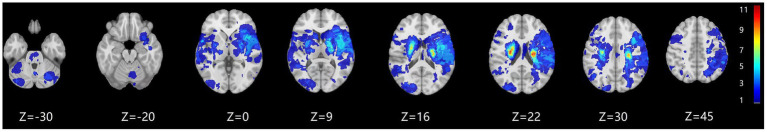
The infarct prevalence map. The prevalence of the infarct is color coded and superimposed on a 1 mm MNI-152 template. The color bar indicates the number of patients with a lesion for each voxel.

The presented study tried to investigated the relationship between the changes in the social behavior and the infarcts (Figure 3), cortical / subcortical structures, cholinergic pathways and white matter. The left cortical infarct was although related to deficit behavioral changes in the univariate analysis, but it was not significant correlation after adjusted for the confounders. And it found that the social behavior was not correlated to involvement of neither the cortical/subcortical structures or cortical cholinergic pathways, except for white matter lesions. The intact SC require a collection of large-scale networks in diverse brain regions. The operation and coordination of such highly distributed brain areas would be impossible without the presence of extensive white matter tracts for the long-range transmission of communication; this neuroanatomical infrastructure might be more crucial for SC than for other cognitive functions that are underpinned by a single structure or a small set of anatomically clustered brain structures (Wang and Olson, 2018).

In the present study, poorer physical status was also correlated with more severe social behavior changes in the subacute phase of ischemic stroke. Clinical factors other than the severity of stroke (as assessed by the NIHSS) may determine the severity of social impairments in stroke patients. Poorer physical function is significantly correlated with reduced abilities of daily living, which may induce multiple dysfunctions such as memory and executive dysfunctions (McDonald et al., 2019). Because our study mainly included stroke patients with mild residual disabilities, it was a relatively homogeneous sample for studying social behavioral changes in ischemic stroke (Nijsse et al., 2019).

Our study had the following strengths. First, it was a longitudinal and prospective study. Second, we assessed social behavior in patients with mild stroke, who were with mild impairments in motor, cognitive and activities of daily living functioning, logically revealed changes in social behavior after ischemic stroke. Third, we used the advanced neuroimaging analysis techniques on the basis of a multimodal MRI protocol. There were also some limitations in this study. First, the sample size was relatively small. Second, we did not investigated the general cognition on the follow-up. Third, although we had excluded severe aphasia according to NIHSS score, in consideration of language impairments have devastating effects on the behavior and mood of affected individuals (Edelkraut et al., 2022), however, we did not specially assess the relationship of the brain damage that occurred in language-dominant left hemisphere and the negative symptoms of behavioral changes. This need to be improved in the further study.

In conclusion, we found that larger DWMH volume and poorer mRS on follow-up were significantly correlated with deficit behavioral changes in subacute ischemic stroke.

## Data availability statement

The original contributions presented in the study are included in the article/Supplementary material, further inquiries can be directed to the corresponding author.

## Ethics statement

The studies involving humans were approved by Ethics Committee of the Tenth Affiliated Hospital of Southern Medical University (Dongguan People’s Hospital). The studies were conducted in accordance with the local legislation and institutional requirements. The participants provided their written informed consent to participate in this study. Written informed consent was obtained from the individual(s) for the publication of any potentially identifiable images or data included in this article.

## Author contributions

J-FQ: Data curation, Formal analysis, Investigation, Visualization, Writing – original draft. H-HH: Data curation, Formal analysis, Investigation, Resources, Writing – review & editing. J-FL: Formal analysis, Investigation, Methodology, Resources, Writing – review & editing. Y-QZ: Methodology, Resources, Software, Validation, Writing – review & editing. W-YC: Data curation, Formal analysis, Investigation, Resources, Writing – review & editing. LS: Investigation, Methodology, Resources, Validation, Writing – review & editing. LZ: Formal analysis, Investigation, Supervision, Visualization, Writing – review & editing. YK-C: Conceptualization, Supervision, Validation, Visualization Writing – review & editing. Y-SL: formal-analysis, investigation, resources, supervision, writing-review-editing.

## References

[ref1] Adams A. G. Henry J. D. Molenberghs P. Robinson G. A. Nott Z. von Hippel W. (2020). The relationship between social cognitive difficulties in the acute stages of stroke and later functional outcomes. Soc. Neurosci. 15, 158–169. doi: 10.1080/17470919.2019.1668845, PMID: 31526327

[ref2] Adams H. P. Jr. Bendixen B. H. Kappelle L. J. Biller J. Love B. B. Gordon D. L.. (1993). Classification of subtype of acute ischemic stroke. Definitions for use in a multicenter clinical trial. TOAST. Trial of org 10172 in acute stroke treatment. Stroke 24, 35–41. doi: 10.1161/01.str.24.1.35, PMID: 7678184

[ref3] Adams A. G. Schweitzer D. Molenberghs P. Henry J. D. (2019). A meta-analytic review of social cognitive function following stroke. Neurosci. Biobehav. Rev. 102, 400–416. doi: 10.1016/j.neubiorev.2019.03.011, PMID: 30922978

[ref4] Benejam B. Sahuquillo J. Poca M. A. Frascheri L. Solana E. Delgado P.. (2009). Quality of life and neurobehavioral changes in survivors of malignant middle cerebral artery infarction. J. Neurol. 256, 1126–1133. doi: 10.1007/s00415-009-5083-9, PMID: 19288045

[ref5] Chaudhury D. Walsh J. J. Friedman A. K. Juarez B. Ku S. M. Koo J. W.. (2013). Rapid regulation of depression-related behaviours by control of midbrain dopamine neurons. Nature 493, 532–536. doi: 10.1038/nature11713, PMID: 23235832 PMC3554860

[ref6] Dai S. Piscicelli C. Lemaire C. Christiaens A. Schotten M. T. Hommel M.. (2022). Recovery of balance and gait after stroke is deteriorated by confluent white matter hyperintensities: cohort study. Ann. Phys. Rehabil. Med. 65:101488. doi: 10.1016/j.rehab.2021.101488, PMID: 33450367

[ref7] Dickie D. A. Gardner K. Wagener A. Wyss A. Arba F. Wardlaw J. M.. (2020). Cortical thickness, white matter hyperintensities, and cognition after stroke. Int. J. Stroke 15, 46–54. doi: 10.1177/1747493019851291, PMID: 31088224

[ref8] Edelkraut L. López-Barroso D. Torres-Prioris M. J. Starkstein S. E. Jorge R. E. Aloisi J.. (2022). Spectrum of neuropsychiatric symptoms in chronic post-stroke aphasia. World. J. Psychiatry 12, 450–469. doi: 10.5498/wjp.v12.i3.450, PMID: 35433325 PMC8968505

[ref9] Fazekas F. Kleinert R. Offenbacher H. Schmidt R. Kleinert G. Payer F.. (1993). Pathologic correlates of incidental MRI white matter signal hyperintensities. Neurology 43, 1683–1689. doi: 10.1212/wnl.43.9.1683, PMID: 8414012

[ref10] Fazekas F. Schmidt R. Scheltens P. (1998). Pathophysiologic mechanisms in the development of age-related white matter changes of the brain. Dement. Geriatr. Cogn. Disord. 9, 2–5. doi: 10.1159/000051182, PMID: 9716237

[ref11] Filley C. M. Fields R. D. (2016). White matter and cognition: making the connection. J. Neurophysiol. 116, 2093–2104. doi: 10.1152/jn.00221.2016, PMID: 27512019 PMC5102321

[ref12] Gunaydin L. A. Grosenick L. Finkelstein J. C. Kauvar I. V. Fenno L. E. Adhikari A.. (2014). Natural neural projection dynamics underlying social behavior. Cells 157, 1535–1551. doi: 10.1016/j.cell.2014.05.017, PMID: 24949967 PMC4123133

[ref13] Henry J. D. von Hippel W. Molenberghs P. Lee T. Sachdev P. S. (2016). Clinical assessment of social cognitive function in neurological disorders. Nat. Rev. Neurol. 12, 28–39. doi: 10.1038/nrneurol.2015.229 26670297

[ref14] Kana O. Kayo N. Tomoko K. Shogo S. Kouichi H. Kobayashi K. (2021). Different cholinergic cell groups in the basal forebrain regulate social interaction and social recognition memory. Sci. Rep. 11:13589. doi: 10.1038/s41598-021-93045-7, PMID: 34193944 PMC8245640

[ref15] Kertesz A. Davidson W. Fox H. (1997). Frontal behavioral inventory: diagnostic criteria for frontal lobe dementia. Can. J. Neurol. Sci. 24, 29–36. doi: 10.1017/s0317167100021053 9043744

[ref16] Kertesz A. Nadkarni N. Davidson W. Thomas A. W. (2000). The frontal behavioral inventory in the differential diagnosis of frontotemporal dementia. J. Int. Neuropsychol. Soc. 6, 460–468. doi: 10.1017/s1355617700644041 10902415

[ref17] Kim W. S. H. Luciw N. J. Atwi S. Shirzadi Z. Dolui S. Detre J. A.. (2022). Associations of white matter hyperintensities with networks of gray matter blood flow and volume in midlife adults: a coronary artery risk development in young adults magnetic resonance imaging substudy. Hum. Brain Mapp. 43, 3680–3693. doi: 10.1002/hbm.25876, PMID: 35429100 PMC9294299

[ref18] Kim J. T. Park M. S. Yoon G. J. Jung H. J. Choi K. H. Nam T. S.. (2011). White matter hyperintensity as a factor associated with delayed mood disorders in patients with acute ischemic stroke. Eur. Neurol. 66, 343–349. doi: 10.1159/000332585, PMID: 22095210

[ref19] Malloy P. Grace J. (2005). A review of rating scales for measuring behavior change due to frontal systems damage. Cogn. Behav. Neurol. 18, 18–27. doi: 10.1097/01.wnn.0000152232.47901.88, PMID: 15761273

[ref20] McDonald S. Dalton K. I. Rushby J. A. Landin-Romero R. (2019). Loss of white matter connections after severe traumatic brain injury (TBI) and its relationship to social cognition. Brain Imaging Behav. 13, 819–829. doi: 10.1007/s11682-018-9906-0, PMID: 29948905

[ref21] Nijsse B. Spikman J. M. Visser-Meily J. M. de Kort P. L. van Heugten C. M. (2019). Social cognition impairments in the long term post stroke. Arch. Phys. Med. Rehabil. 100, 1300–1307. doi: 10.1016/j.apmr.2019.01.023, PMID: 30831095

[ref22] Ozzoude M. Varriano B. Beaton D. Ramirez J. Holmes M. F. Scott C. J. M.. (2022). Investigating the contribution of white matter hyperintensities and cortical thickness to empathy in neurodegenerative and cerebrovascular diseases. GeroScience 44, 1575–1598. doi: 10.1007/s11357-022-00539-x, PMID: 35294697 PMC9213606

[ref23] Phillips L. H. Scott C. Henry J. D. Mowat D. Bell J. S. (2010). Emotion perception in Alzheimer’s disease and mood disorder in old age. Psychol. Aging 25, 38–47. doi: 10.1037/a0017369, PMID: 20230126

[ref24] Powers W. J. Rabinstein A. A. Ackerson T. Adeoye O. M. Bambakidis N. C. Becker K.. (2019). Guidelines for the early management of patients with acute ischemic stroke: 2019 update to the 2018 guidelines for the early management of acute ischemic stroke: a guideline for healthcare professionals from the American Heart Association/American Stroke Association. Stroke 50, e344–e418. doi: 10.1161/STR.0000000000000211, PMID: 31662037

[ref25] Qu J. F. Chen Y. K. Luo G. P. Zhao J. H. Zhong H. H. Yin H. P. (2018). Severe lesions involving cortical cholinergic pathways predict poorer functional outcome in acute ischemic stroke. Stroke 49, 2983–2989. doi: 10.1161/STROKEAHA.118.023196 30571427 PMC6257508

[ref26] Qu J. F. Zhong H. H. Liang W. C. Chen Y. K. Liu Y. L. Li W. (2022a). Neuroimaging risk factors for participation restriction after acute ischemic stroke: 1-year follow-up study. J. Investig. Med. 70, 363–368. doi: 10.1136/jim-2020-001675, PMID: 34611033 PMC8819659

[ref27] Qu J. F. Zhou Y. Q. Liu J. F. Hu H. H. Cheng W. Y. Lu Z. H.. (2022b). Right cortical infarction and a reduction in putamen volume may be correlated with empathy in patients after subacute ischemic stroke-a multimodal magnetic resonance imaging study. J. Clin. Med. 11:4479. doi: 10.3390/jcm11154479, PMID: 35956096 PMC9369598

[ref28] Santamaría-García H. Ogonowsky N. Baez S. Palacio N. Reyes P. Schulte M.. (2022). Neurocognitive patterns across genetic levels in behavioral variant frontotemporal dementia: a multiple single cases study. BMC Neurol. 22:454. doi: 10.1186/s12883-022-02954-1, PMID: 36474176 PMC9724347

[ref29] Selden N. R. Gitelman D. R. Salamon-Murayama N. Parrish T. B. Mesulam M. M. (1998). Trajectories of cholinergic pathways within the cerebral hemispheres of the human brain. Brain 121, 2249–2257. doi: 10.1093/brain/121.12.2249, PMID: 9874478

[ref30] Tziaka E. Christidi F. Tsiptsios D. Sousanidou A. Karatzetzou S. Tsiakiri A.. (2023). Leukoaraiosis as a predictor of depression and cognitive impairment among stroke survivors: a systematic review. Neurol. Int. 15, 238–272. doi: 10.3390/neurolint15010016, PMID: 36810471 PMC9944578

[ref31] Wang K. Hoosain R. Lee T. Meng Y. Fu J. Yang R. (2006). Perception of six basic emotional facial expressions by the Chinese. J. Cross Cult. Psychol. 37, 623–629. doi: 10.1177/0022022106290481

[ref32] Wang Y. Olson I. R. (2018). The original social network: white matter and social cognition. Trends Cogn. Sci. 22, 504–516. doi: 10.1016/j.tics.2018.03.005, PMID: 29628441 PMC5960431

[ref33] Yang L. Shu J. Yan A. Yang F. Xu Z. Wei W. (2022). White matter hyperintensities-related cortical changes and correlation with mild behavioral impairment. Adv. Med. Sci. 67, 241–249. doi: 10.1016/j.advms.2022.06.002, PMID: 35780532

[ref34] Yanina V. Barbara Y. Beccaria J. P. Murer M. G. Belforte J. E. (2017). Compulsive social behavior emerges after selective ablation of striatal cholinergic interneurons. J. Neurosci. 37, 2849–2858. doi: 10.1523/JNEUROSCI.3460-16.2017, PMID: 28193688 PMC6596731

[ref35] Zhang F. Ping Y. Jin X. Hou X. Song J. (2023). White matter hyperintensities and post-stroke depression: a systematic review and meta-analysis. J. Affect. Disord. 320, 370–380. doi: 10.1016/j.jad.2022.09.166, PMID: 36209775

[ref36] Zhao Q. Neumann D. L. Cao X. Baron-Cohen S. Sun X. Cao Y.. (2018). Validation of the empathy quotient in mainland China. J. Pers. Assess. 100, 333–342. doi: 10.1080/00223891.2017.1324458, PMID: 28537758

