# Supplementary material for: Larger deep white matter hyperintensity volume correlates with more severe social behavioral changes in patients with subacute ischemic stroke
Source: Front Aging Neurosci. 2023 Nov 22;15:1259690. doi: 10.3389/fnagi.2023.1259690 (PMC10702571; doi:10.3389/fnagi.2023.1259690)
Supplement: Supplementary file 1 [file Table_1.DOCX]

Supplemental Table. Correlation analysis between total brain structure volumes and Frontal Behavioral Inventory scores

| Variables | Volume(ml) |  | Deficit behavior | |  | Positive behavior | |
| --- | --- | --- | --- | --- | --- | --- | --- |
|  |  |  | r | p |  | r | p |
| ICV | 1438.82±126.23 |  | 0.008 | 0.946 |  | -0.023 | 0.843 |
| Brain parenchyma | 1095.38±155.35 |  | 0.017 | 0.879 |  | 0.051 | 0.652 |
| Hippocampus | 6.81±0.63 |  | 0.000 | 0.999 |  | 0.098 | 0.386 |
| Amygdala | 3.72±0.38 |  | 0.025 | 0.828 |  | -0.014 | 0.905 |
| Thalamus | 11.77±1.67 |  | -0.078 | 0.493 |  | 0.126 | 0.265 |
| Caudate | 6.8±0.91 |  | 0.064 | 0.575 |  | 0.118 | 0.298 |
| Putamen | 10.47±1.35 |  | -0.153 | 0.175 |  | 0.108 | 0.342 |
| Pallidum | 3.09±0.44 |  | -0.058 | 0.607 |  | 0.123 | 0.278 |
| Accumbens | 1.07±0.13 |  | -0.017 | 0.880 |  | 0.149 | 0.187 |
| Hypothalamus | 0.69±0.08 |  | 0.021 | 0.855 |  | -0.047 | 0.676 |
| Midbrain | 5.92±0.6 |  | -0.042 | 0.714 |  | 0.056 | 0.623 |
| Pons | 13.86±1.81 |  | -0.021 | 0.851 |  | 0.045 | 0.695 |
| Medulla | 4.29±0.47 |  | -0.027 | 0.813 |  | 0.035 | 0.755 |
| SCP | 0.22±0.03 |  | -0.011 | 0.922 |  | -0.013 | 0.909 |
| Cerebellum | 132.74±12.22 |  | 0.004 | 0.974 |  | -0.053 | 0.643 |

Note: ICV: intracranial volume, SCP: superior cerebellar peduncle.
